# Military veterans’ perspectives on using music to manage chronic pain: themes from the feasibility and acceptability of music imagery and listening interventions for analgesia study

**DOI:** 10.3389/fneur.2025.1613220

**Published:** 2025-08-06

**Authors:** Kristin Maya Story, Sally Wasmuth, Johanne M. Belkiewitz, Claire Whalen, Sheri L. Robb, Dawn M. Bravata, Matthew J. Bair

**Affiliations:** ^1^VA Center for Health Information and Communication, Indianapolis, IN, United States; ^2^Department of Medicine, Indiana University School of Medicine, Indianapolis, IN, United States; ^3^Department of Occupational Therapy, Indiana University School of Health & Human Sciences, Indianapolis, IN, United States; ^4^Indiana University School of Nursing, Indianapolis, IN, United States; ^5^Regenstrief Institute, Indianapolis, IN, United States

**Keywords:** chronic pain, music therapy, imagery, veterans, interviews, acceptability, pilot trial

## Abstract

**Introduction:**

Chronic pain conditions are common in military veterans, often leading to disability, psychological distress and high healthcare utilization. An interdisciplinary approach, informed by a biopsychosocial model, is recommended for patients with chronic pain. Music-based interventions have shown improvements in patients with pain, but results are inconsistent and most studies have concentrated on acute pain and in-person delivery.

**Objective:**

The Feasibility and Acceptability of Music and Imagery for Analgesia (FAMILIA) explored the use of two telehealth delivered music interventions for chronic pain. As part of FAMILIA we conducted interviews to assess the veteran experience and acceptability of the music interventions.

**Methods:**

Semi-structured interviews were conducted with veterans who participated in either self-directed, independent music listening or a therapist-delivered music and imagery intervention in a three-arm randomized controlled trial. All interventions were conducted by board certified music therapists over a HIPAA approved telehealth platform. Interviews were conducted by team members who did not deliver the intervention and included questions about the intervention(s), delivery format, barriers and facilitators to study participation.

**Results:**

Sixteen interviews were recorded, transcribed, and deidentified for analysis. The research team identified ten themes, drawn from the veterans’ experiences from pre to post intervention about the acceptability, motivation for joining, challenges, and perceived benefits of the telehealth-delivered music interventions.

**Conclusion:**

Veteran patients found FAMILIA acceptable and endorsed using music listening or music and imagery as a non-pharmacological support for management of chronic pain and accompanying psychological symptoms.

**Clinical trial registration:**

Clinicaltrial.gov, Identifier NCT05426941.

## Introduction

Pain is a global issue linked to morbidity and disability (1). Chronic pain affects 40–70% of Veterans and is a leading cause of disability, with substantial negative impact on Veterans’ lives (2). Musculoskeletal pain conditions are especially common, accounting for two-thirds of all clinical visits for pain (3). Chronic pain is frequently accompanied by psychological comorbidity that adds to patient suffering and complicates treatment. Veterans with pain and post-traumatic stress disorder (PTSD) demonstrate greater pain, depression, and healthcare utilization, and patients with comorbid pain and depression have worse outcomes than patients with pain or depression alone (4, 5). Medications including opioids are commonly used to treat chronic pain yet have side effects and modest benefits. There is a pressing need to provide effective, non-pharmacological treatment to Veterans with chronic musculoskeletal pain. The VA recommends a comprehensive, interdisciplinary approach to chronic pain within a continuum of care that is informed by a biopsychosocial model (6).

Music therapy interventions address biopsychosocial outcomes and offer promising non-pharmacological options for managing pain and accompanying psychological distress. These interventions can be categorized into active (music making) and receptive (music listening) approaches, with most of the pain research focusing on receptive interventions (7, 8). Music listening (ML) studies have shown significant reductions in pain, emotional distress, and opioid use, but results are inconsistent, and although some studies have examined chronic pain, most research has concentrated on acute pain (9–13). Many of these studies involved recorded ML, with variable outcomes likely due to the lack of theoretical frameworks guiding music selection and delivery, as well as the absence of education to encourage independent music use. Typically, the published ML intervention studies required minimal or no interaction with a music therapist and were designed for independent listening by the patient (8, 10). However, patients with complex pain and psychological issues might benefit from more intensive and interactive ML, which includes ongoing support, symptom monitoring, and additional therapeutic components like imagery and verbal processing, to potentially enhance the effectiveness of ML. For example, music and imagery (MI) is a receptive music therapy intervention that combines ML with imagery and verbal processing to meet diverse patient needs, including those with PTSD, cancer, mood disorders, and chronic pain (14–18).

The COVID-19 pandemic accelerated the use of telehealth for various chronic conditions, including chronic pain, yet there is limited research on virtually delivered music therapy. A 2021 survey of Veterans Administration creative arts therapists revealed that 76% had conducted virtual sessions, with 74% delivering more than 50 sessions in one year (19). Although telehealth potentially offers increased access, convenience, and continuity of care for Veterans, there is a need for rigorous research to evaluate the feasibility, acceptability, and efficacy of virtual music therapy interventions. The Feasibility and Acceptability of Music Imagery and Listening Interventions for Analgesia (FAMILIA) Study (ClinicalTrials.gov NCT05426941) addresses limitations in music intervention research for chronic pain and knowledge gaps on virtually delivered music therapy. FAMILIA assessed two types of telehealth delivered music interventions (MI and ML) for Veterans with chronic musculoskeletal pain and evaluated feasibility, acceptability, pain relief, and related outcomes. This manuscript reports acceptability findings of FAMILIA, assessed through qualitative analysis of Veterans’ reported experiences of the music interventions. Feasibility metrics are reported in another paper. Through semi-structured interviews with FAMILIA participants, we explored: (1) Veterans’ experiences with both interventions; (2) aspects of the interventions and virtual delivery that Veterans perceived to be most or least helpful or liked; and (3) barriers and facilitators to study participation.

## Methods

### Music interventions

#### Music listening (ML)

Each participant randomized to ML had one meeting with a board-certified music therapist to identify: musical tastes, preferences and activities; musical memories and social influences; and relationships between music, health, and quality of life (20). From this meeting, the therapist compiled an electronic playlist for the Veteran to listen to during the 12-week treatment period. Participants were free to listen to other music of their choice in addition to the playlist and were asked to maintain a music listening log detailing: how long they listened, what they listened to, and any reactions to the music (e.g., benefits, triggers). Music listening time was not prescribed, but participants were encouraged to keep track of their listening during the 12 weeks. Participants were asked to mail or scan and upload their music logs at study completion. A member of the research team checked in with ML participants weekly for the first three weeks following their initial session, then bi-weekly following the 1-month assessment to monitor safety, adherence (i.e., time listening to music), and identify other issues. The rationale for including the ML intervention was to explore whether there is clinically meaningful change in measures and perceived benefits from a less interactive, less intensive music intervention.

#### Music and imagery (MI)

The MI intervention is derived from The Bonny Method of Guided Imagery and Music (21) and structures a depth-oriented music psychotherapy method into a more contained supportive method that focuses on one self-identified issue or area of need that can be explored within a 45–60-min individual session. Under the umbrella term of Guided Imagery and Music, there are many interventions that combine music and imagery to target symptom management, functional health, and well-being outcomes. The MI intervention for this study was the supportive MI methods from the Continuum Model of MI developed by Summer (22).

Each participant randomized to MI received up to 8, 45-min weekly sessions over 8–12 weeks. MI sessions were delivered by a board-certified music therapist with specialized training in the Continuum Model of MI. Sessions entail four steps:

1. Slow down and recognize current condition (e.g., presence of pain, tension, emotional states), and desired condition (e.g., decreased pain, relaxation, enhanced positive mood). Imagery generated by the Veteran is used to aid in the description of the current and desired state.
2. Choose music together from the Veteran’s pool of music that will help care for what needs attention. This is accomplished through a slow intentional process of comparison and discussion of their music and finding the best fit for the desired state.
3. Listen to final music selection together with optional drawing, journaling, or movement to enhance the experience. The optional creative modalities are used to help explore Veteran-generated imagery, however it can also be explored through verbal discussion to deepen the experience. The chosen music is repeated to enhance the desired state.
4. Identify homework: The music therapist and Veteran identify specific ways to engage with the music and imagery between sessions (e.g., set aside time to listen to same piece, or explore images with further drawing or journaling).

The primary aim in MI for chronic pain management is to address participants’ relationship to pain, their ability to interact adaptively with chronic pain, and to use supportive and accessible tools (e.g., music to self-regulate through relaxation, distraction, increased connection to inner resources) to manage pain and related psychological symptoms (e.g., depression, anxiety).

A brief description of MI and ML interventions are outlined in Table 1.

**Table 1:** Study groups description.

Music listening	Music imagery	Usual care
The Veteran meets with a music therapist (one 45-min virtual meeting) to identify musical tastes, preferences, activities, musical memories, and social influences, and to learn about the relationship between music health and quality of life. From this meeting a CD or electronic playlist is made for the Veteran to listen to during the 12-week treatment period. The Veteran is asked to record how long they listen, what they listen to, and any reactions to the music (benefits, and triggers) in a listening log. The Veteran receives a check-in by study staff by phone or email, up to 8 times during 12-week period to touch base on potential problems with music listening and identify any technical issues.	The Veteran participates in up to 8, 45-min virtually delivered music therapy sessions over 8–12 weeks. The steps of MI involve: “Checking in” to identify Veteran’s current state in terms of pain and mood and to create an image of desired state (10 min). Veteran and music therapist choose music together from Veteran’s playlists to enhance the desired state (5 min). Listen to music together with additional optional creative processing (for example drawing, creative writing, movement) to bring out a creative representation of the image/desired state (15 min). Identify ways to work with the music and imagery during the week with repeated music listening (15 min) Listening Log – In between music and imagery sessions, the Veteran is asked to record how long they listen to music, what they listen to, and any reactions to the music (benefits, and triggers) in a listening log.	The Veteran is not asked to stop current analgesics (pain medications) or non-pharmaceutical treatments (for example physical therapy) provided according to usual standards of care through their normal provider.

#### Study methods

Details of FAMILIA background, study design, outcomes of interest, and methods can be found in the published protocol (23). In brief, 60 Veterans with musculoskeletal chronic pain were randomized to one of three study arms: 12 weeks of independent ML following one telehealth meeting with a music therapist, eight weekly supportive MI sessions (over a 12-week window) facilitated through telehealth with a board-certified music therapist, or usual care. All intervention sessions were individual 1:1 sessions. Veterans randomized to the music interventions were asked to complete music listening logs that detailed how often they listened to music independently between meetings. During the final assessment meeting with research staff, participants were invited to be interviewed, and if they agreed, were interviewed by the principal investigator and the study therapist who did not deliver the intervention to them. The semi-structured interview included questions designed to elicit Veteran’s experiences of the intervention(s), the virtual delivery format, and barriers and facilitators to study participation (interview guide included in Supplementary Appendix 1). Interviews were conducted through Microsoft Teams video calls and, with the Veteran’s permission, were recorded, transcribed, and de-identified. Interviews lasted 30–45 min. FAMILIA was reviewed and approved by Indiana University’s Institutional Review Board (#12794).

### Data analysis

We used the six steps of thematic analysis to assess interview data from Veterans (32), which include: familiarization, generation of codes, combining codes into themes, reviewing themes, defining and naming themes, and reporting of findings.

Three research members (MB, KMS, SW) independently read through two interviews and collaborated in the generation of 16 initial codes, paying special attention to data related to the experience of interventions and delivery and barriers/facilitators to study participation. Continued familiarization with the data resulted in adding one code and combining others. Following revision of the initial codes, three research members (JB, KS, CW) read through all transcripts, independently coding them, meeting to discuss and revise the codes, and returning to the transcripts as needed. This iterative process resulted in eight final themes, discussed below. Microsoft Excel was used to organize qualitative data and facilitate coding. When all 16 transcripts had been coded, themes were shared with other members of the research team for feedback (DB, MB, SW). The data and final themes reflected the temporal process of study participation and were therefore organized under the three overarching categories of ‘pre-intervention’, ‘during intervention’, and ‘post-intervention.’

## Results

From the 45 participants randomized to one of the music intervention arms, we interviewed 16 Veterans, including participants treated by each of the music therapists and from each intervention group. Interviewed Veterans had participated in the ML (*N* = 7) or MI (*N* = 9) interventions. We included more Veterans from the MI group because of the greater number of participants randomized to that intervention. Characteristics of the Veterans interviewed are depicted in Table 2.

**Table 2:** Participant characteristics.

Characteristic	*N* = 16
Age median (range)	57 (36–74)
Male Sex *N* (%)	10 (62.5)
Race/ethnicity *N* (%)	
American Indian or Alaska Native	1 (6.3)
Asian	0
Black or African American	4 (25.0)
White or Caucasian	10 (62.5)
Other	1 (6.3)
Hispanic or Latino	0
Marital Status *N* (%)	
Married	8 (50.0)
Divorced	6 (37.5)
Widowed	1 (6.3)
Never married	1 (6.3)
Employment *N* (%)	
Employed	6 (37.5)
Retired	6 (37.5)
Unable to work	4 (25.0)
Education *N* (%)	
4-year college degree	7 (43.8)
2-year college degree or some college	6 (37.5)
Post-graduate degree	2 (12.5)
Highschool or GED	1 (6.3)

### Pre-intervention

Two themes emerged as factors that existed prior to Veterans’ study participation. The first was their existing relationship with music and the ways in which that relationship impacted Veterans’ interest in and experience of study participation. The second was an existing motivation to seek out non-pharmacological pain management options. Due to this motivation, music therapy presented as an appealing opportunity.

#### Existing relationship with music

Participants came to the study with an existing relationship with music, which included strong memories, associations, and preconceived ideas about music. Many mentioned that music was already a regular part of their routine. “*I listen to music all day at work while I’m working anyways and in the car driving, so, I mean, that was just part of my normal daily routine”* (ML 355). They also previously used music therapeutically, though their awareness of that utility was minimal, or they had a vague idea of how they used music to make them feel better. “*I used music for a long time. I did not know anything about it. I just knew that music soothed me”* (MI 159).

Given their existing relationship with music, Veterans came to the study with preconceived ideas about whether music might be helpful, or how it might be used, *“…it was very interesting. I had a preconceived notion going in that she’s [the music therapist] going to have this music list of things that studies show helps and it wasn’t that way at all… So, that was a neat process and blew my preconceptions away”* (MI 271). The same Veteran also spoke about using that pre-existing relationship with music to help manage pain. “*And it ended up opening my mind and bringing me back to years and years ago when I listened to music quite a bit because some of the songs of my youth that helped. It helped me remember those prior-to-pain times and, and that helped identify the pain and then push it away to where back in those times, I’m not hurting. I’m not in pain”* (MI 271).

The connection between music and memories from different stages of their life also speaks to the strong connection to their identity that was enhanced during the music interventions. Veterans, as in the quotation above, spoke of music connecting them to a different time of their life before they had pain, or before being someone with chronic pain became a part of their identity. Some Veterans spoke of a deeper connection music has to their identity. *“It kind of made me think about some of those good times, or you know even if it wasn’t a good time, that the music is a central part of who I am”* (ML 408).

#### Motivation to find non-pharmacological pain management options

Veterans were motivated to find non-pharmacological options for pain management. They spoke of not wanting to take medication or having invasive procedures. *“It’s great because I just feel like anything that you can do that do not involve medicine or cutting or surgeries, it’s always a better option”* (MI 206). There were some who could not take medication while they were working, but for others, it was a preference. *“Well, like I say, with anything, you know, it’s a chance and I was looking for another way to deal with my pain. I’m not a pill person”* (MI 200). There was also a desire for something more than what medical interventions could provide. *“We need more than a doctor’s visit or MRI or pain injection, you know or a pill. We need some type of therapy that’s going to help us look differently at ourself and free ourself. So, we can move to a different level”* (MI 284).

### During intervention

Within this category Veterans described facilitators and barriers to study participation. These are organized into two facilitator themes and three barrier themes as described below.

#### Intervention and delivery flexibility

Veterans appreciated the flexibility of both music therapy interventions and the virtual mode in which the interventions were delivered.

Although the research protocol was highly structured, the intervention manual allowed for various forms of Veteran self-expression. *“I found myself dancing and nodding my head but she never said anything like you cannot dance or anything like that.”* (MI 206). During independent ML, Veterans appreciated flexibility of being able to choose what to listen to, even if it was a change from the initial playlist. *“In the end, the type I told her wasn’t really the type I ended up feeling the best with. The first two weeks I just did the ones that we created and after that I took another block [of music] and then I started pulling music that I felt the best with from both of ‘em”* (ML 165).

Veterans appreciated being able to participate through telehealth. Time management was often mentioned as a factor that played into preference for telehealth. *“Virtual is always going to be a thing when you are pressed for time ‘cause I feel like sometimes life gets busy. So, there’s been time when I got off work and I literally ran upstairs you know, so virtual is good for that, far as time wise”* (MI 206). In addition to reducing travel time, telehealth provided a better environment for one Veteran who did not like going to the VA medical center. *“I like virtual, it’s like I think that I get the same benefit, you know, I mean, at least in my humble opinion. It cuts down on travel time ‘cause it would take me 40 min to get to the VA and then dealing with the stress of the VA. I think it would be such a massive distraction ‘cause I do not like going to the main VA. I hate the main VA”* (MI 199).

#### Rapport with therapist

Veterans spoke about being comfortable and supported by the therapists. Several expressed not wanting the sessions to end. *“She was definitely inspiration with me. You know, made me feel relaxed. Made me feel like, you could do this, do not worry about it… I can remember as we were wrapping up how emotional it was, more bittersweet, you know…she just kind of worked with me and, we just worked together”* (MI 200).

#### Time burden to complete study measures

Perceived burdens to study participation were the time required to fill out the music listening logs each time they listened. Many suggestions were offered by participants on how to make the music listening log less burdensome, such as using an electronic log instead of a paper version and simplifying log content to include music genres instead of recording each individual song. Additionally, some Veterans reported a time burden associated with the study assessments conducted at four timepoints (i.e., baseline, 1, 3, and 4-months).

#### Decreased presence and social interaction

Although some appreciated the flexibility of virtual meetings, others were aware of how the experience may have been different in person. Veterans spoke about the quality of their interactions with the music therapists in a virtual meeting versus in-person. *“I probably would have talked more if it was in person”* (MI 159). One Veteran felt that the virtual environment involved distractions. *“It would have been nice to like done that in person. It makes it easier for people like me with a TBI [Traumatic Brain Injury] to relate, you know, I may get distracted and try to be doing something else while I’m trying to carry conversation on. It’s so easy to just get distracted and when I know that the person’s right there in front of me, it’s easier”* (ML 243). Another Veteran spoke of social isolation not being addressed when the sessions are virtual. *“Obviously, it’s convenient to stay at home but I think there probably should be an in-person option too because, I mean, I can only speak for myself but I’m kind of isolated socially, so it would be nice to come in”* (MI 321).

#### Technical issues

Technical issues were often mentioned as an annoyance but typical and expected. Some mentioned that the music quality was reduced when being shared virtually. Veterans were aware that spending time to troubleshoot technology took away from time spent on the music intervention.

### Post-intervention

Veterans described lasting benefits, endorsing music therapy to other Veterans, and some remaining challenges following participation in the music therapy interventions. These data are organized into three themes as described below.

#### Biopsychosocial benefits

Physical benefits included increased relaxation, pain relief, improved sleep, and greater ability to participate in daily activities. Notable quotations, such as this participant, spoke of decreasing pain medication, *“this is something that they put out there to me that I do not have to go take a pain pill every time. It definitely gives me some relief. I just say that that’s the best thing that could have came out of this for me. I do not take as many pain pills”* (MI 200).

Reported psychological benefits included improved mood or mental outlook. There were examples of this with ML, *“But when I’m kind of down or depressed, maybe because of pain and environmental situations, then I start listening to the music and the music does take my mind off of what’s happening, you know? It’s a totally different focus.”* (ML 221). Veterans also reported improved mood following individual MI sessions with a therapist, *“yeah, I got into the groove of things and I was actually kind of like mentally happy after each one”* (MI 321).

#### Increased knowledge of emotion regulation through music

One Veteran specifically articulated their understanding of the different functions of the music versus the imagery, *“the music by itself, it’s a very good calm, something to calm you down. Something to help you relax and the imagery helps you to bring whatever the issues are to the surface and it helps you to like bring it out a little bit more and you may see things in a way that you did not see before”* (MI 159).

Their awareness and understanding of how to use music changed because of participating in the study. *“I just never thought that music could make me, you know, just unwind like that. I mean, I’ve listened to music all my life, but I just did not like, you know, use it to help relieve pain and when I did that, when I put that with that, it just kind of made sense to me”* (MI 200).

Increased awareness of positive and negative associations to music led to an increased ability to choose appropriate music for a given situation. Veterans were aware of the negative associations that would emerge during ML when it was connected to specific memories. One Veteran spoke about the effect of listening to a John Coltrane song that was written in memory of a church bombing that occurred when this Veteran was younger and living in the South when segregation was prominent. The quote also speaks to music’s connection to identity. *“It did not bring me into a depressive state but it just kind of, you know, oh man that was terrible, you know, sad but it wasn’t something that lingered. It’s part of who I am, I learn to deal with it”* (ML 408). Another Veteran was aware of the difficult memories music brought up but chose different music when that happened. *“Some of the music brought up some of my past that wasn’t extremely easy to deal with. So, I chose to change the channel”* (ML 161). One Veteran thought that listening to music from a certain time would be helpful but was not. *“Sometimes, you hear a song that kind of made ya sad. Like I said, I lost my wife back in the day to cancer. She had certain songs she liked and when I put them on, I thought it would help. It did not. So, it all came out a lot more than I expected”* (ML 165).

Veterans reported using music to slow down, relax, and regulate responses to stress. *“When I find myself distracted, I’ve gone back to the techniques that I learned during the study…Just being able to calm myself from a stressful situation through the use of music”* (ML 161). Conversely, it was also used to energize and motivate some Veterans. *“I do suffer from depression and stuff like that too. It kicks in back and forth and like I said, when I listen to a couple of my playlists, it gives me that I will not quit attitude. I cannot quit”* (ML 221).

#### Endorsement and continuation

More than 87% (14/16) of Veterans reported continued use of techniques they learned during the intervention period. *“I was able to take what I’ve learned and apply that with some of the music selections of my choice while I’m, you know, sitting in the office or even driving home. And I’m in the car for roughly three hours plus a day”* (MI 271).

Veterans endorsed the use of ML and MI with other Veterans with chronic pain as well as those with other conditions. Veterans with mental health issues, PTSD, and housing insecurity were specifically mentioned. *“Yeah, these Vets, we need this. I mean, it’s quick to say let us do medicine, you know, pill wise, but I’d say there is an alternative and the music therapy is definitely one for me”* (MI 200) Table 3.

**Table 3:** Themes by study process time.

Study time period	Theme	MI, ML, or Shared across groups
Pre-intervention	Existing relationship to music	Shared theme
	Motivation to find non-pharmacological pain management options	Shared theme
During intervention: facilitators	Intervention and delivery flexibility	Shared theme
	Rapport with therapist	MI theme
During intervention: barriers	Time burden to complete study measures	Shared theme
	Decreased presence and social interaction	Shared theme, more prominent in MI group
	Technical issues	MI theme
Post-intervention	Lasting biopsychosocial benefits	Shared theme
	Increased knowledge of emotion regulation through music	Shared theme
	Endorsement and continuation	Shared theme

## Discussion

### Motivation to join and changes in music relationship

Our results indicate that finding additional ways to manage pain, and interest in music were motivations to enroll in FAMILIA. Other studies have documented that Veterans with chronic pain are motivated to find non-pharmacological treatments and are open to complimentary and integrated health modalities (24, 25). Music is increasingly accessed or randomly encountered in everyday life, and previous studies have indicated that music listeners are aware that music affects their mood (26, 27). Many of the Veterans who participated in FAMILIA described themselves as having a relationship or connection to music, but were not always aware of how their relationship to music affected their experiences of chronic pain. Previous studies have documented the association of music with memories of military training, deployment, and returning home (28). The existing relationship with music may be one reason the intervention is accessible and helpful for many.

**Figure 1:**
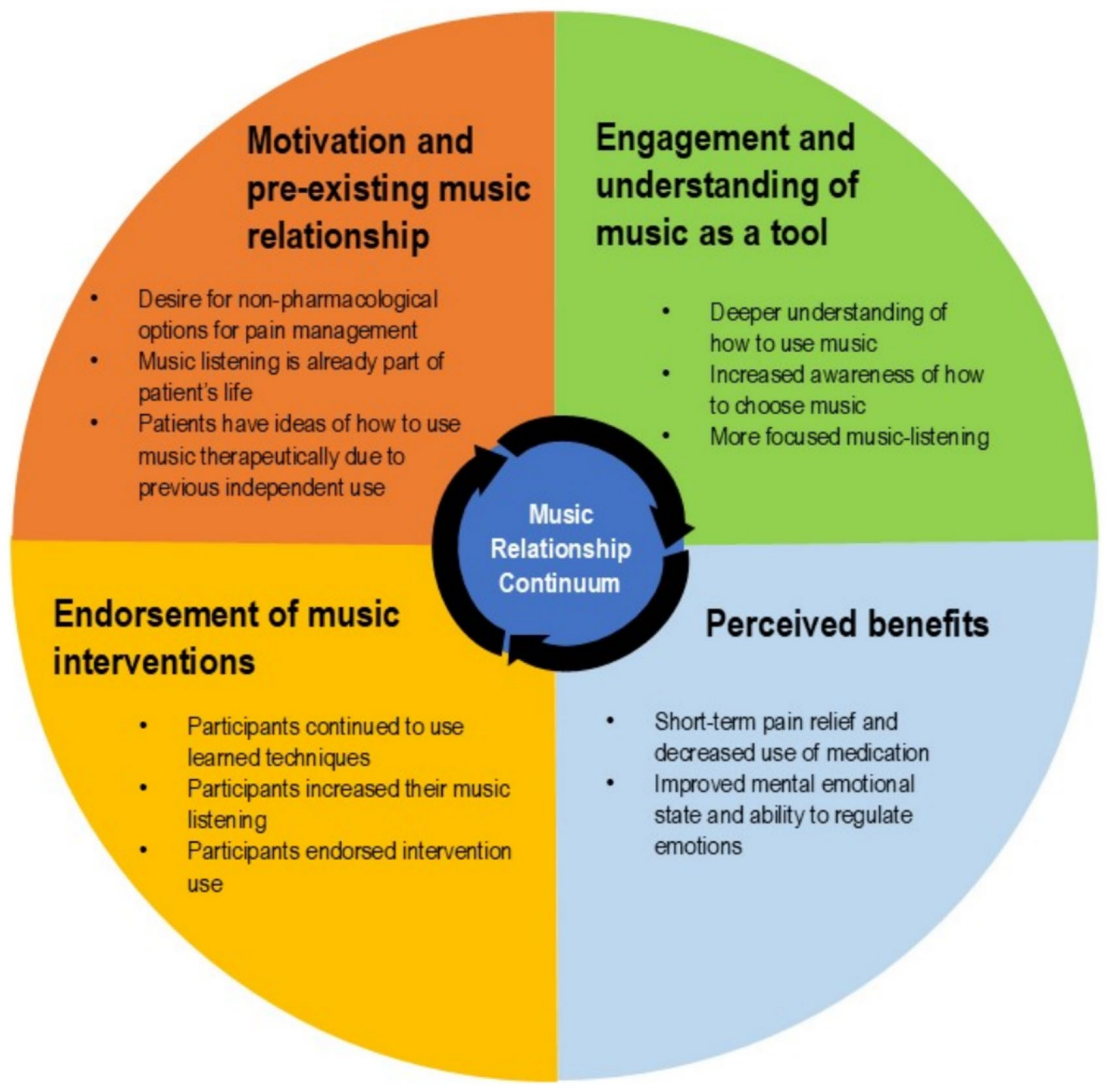
Music relationship continuum.

Veterans from both music interventions expressed that the way they thought about music or chose music changed in some way because of their study participation. In particular Veterans were more aware of the affect of music on their mood, energy, or pain and this increased awareness and intentionality led to continued use. Most people have a relationship to music due to its ubiquity in society and everyday life. Some Veterans have music associations specific to their military careers; but as participants engaged, their understanding of how to use music deepened, leading to more focused music-listening and benefits beyond pure enjoyment. The perceived benefits from engaging in ML or MI reinforced continued use. Veterans reported an increase in listening as well as endorsement of the music interventions for other Veterans with a variety of clinical needs (e.g., pain, mental health, homelessness). This endorsement aligns with previous findings showing that Veterans prefer integrative health approaches. Between 2016 and 2019, their use of complementary therapies increased by 70% and was especially high in Veterans with chronic musculoskeletal pain (25). Figure 1 illustrates this cyclical music relationship as it relates to central themes from the study. Namely that participants come to the study with an existing relationship to music, through study interactions they increase their understanding of music and that relationship, experience the biopsychosocial benefits of music, leading to an increase in music listening and interaction with learned techniques, and that stronger relationship to music is brought into subsequent interactions with music.

### Differences and similarities between the music interventions

We approached the analysis of the music interventions as a whole to gain a better understanding of Veterans’ experiences with both interventions, aspects of the interventions and virtual delivery that Veterans perceived to be most or least helpful or liked, and barriers and facilitators to study participation; however, we noted some differences that emerged from the data. Most often for both groups, music was mentioned as a distraction, but this was especially true of the ML participants who listened to music independently. Unique to MI participants were reports of being more aware of their change in perspective and understanding of their pain, their relationship to pain, or their identity as a person with chronic pain. Centrality of pain, defined as the degree to which an individual views chronic pain as a principal feature of their life experience or identity, is a construct conceptualized from clinical observations that patients with poor pain control focus on their pain more than other aspects of their lives, to the extent of pain being central to their identity (29). Whereas FAMILIA participants from both groups shared about their experiences of reduced pain and interference in their daily lives, only MI participants spoke about a change in identity that would suggest a shift in centrality of pain. Though music was reported as helpful to both groups, it may have served a different function when used for listening and short-term distraction from pain, or as it is in MI where the aim is to enhance inner resources and gain a different perspective of pain.

The MI weekly sessions with a music therapist likely led to a higher level of absorption in the music. Not only was there more repetition than we could guarantee was occurring with the ML group, but the shared music listening with the therapist created a different level of attention on the music. Listening to music independently could likely become background music at times, despite the education around focused listening, but shared listening was more intentional. In addition, the music in combination with the imagery added a different level of attention and absorption. One way to understand absorption is through the Cognitive Vitality Model, a model that theorizes five cognitive mechanisms underlying music-listening interventions (30). The Cognitive Vitality Model recognizes that in music-listening interventions there are a variety of cognitive processes that dynamically interact through the music and the listener’s cognitive state. These processes are linked together, beginning with the automated attention present with most audio stimuli that with active engagement leads to enjoyment, meaning-making, absorption in the experience, and cognitive vitality (strengthened sense of self). The music and the imagery were components that in combination may have contributed to more absorption and cognitive vitality. Both centrality of pain and level of absorption are concepts to explore in further studies.

Participants in both groups spoke about the connection between music, emotions, and memories. Some spoke about music connecting them to a time before they had chronic pain. This connection to the memory of a healthy self has been found in other qualitative studies on music and pain (31). Several Veterans in the ML group spoke about a music association to painful memories. In those moments, they switched to more helpful music. The potential negative associations highlight the importance of having therapist interactions to educate about music choices or when appropriate, use the therapy sessions to support safe exploration of memories and accompanied emotions. This may be especially salient to Veterans and military personnel who have reported positive *and* negative associations of music used during active deployment (28).

### Implication for dosage and relief

Some Veterans reported that the pain relief from listening to music did not last long. We did not track exact times on the duration of pain relief, but some mentioned a few hours, or just stated that it was short-term. When music is used to distract from pain, more frequent exposure may be required. When long-term results were mentioned, they were either because they had learned to use music differently and were continuing to use techniques learned, or they were in the MI group and discussed a change in perspective about their ability to live with chronic pain and accomplish goals. It is important to highlight that although both music interventions had a duration of 12 weeks, ML participants had one meeting with a music therapist and MI had up to eight meetings. We introduced the interventions as less intensive (ML) and more intensive (MI), but one could also view it as one versus eight doses of music therapy.

Previous studies have identified an association between ML and improvements in self-reported pain, but we lack evidence regarding pain relief duration, or how music might be used for on-going symptom management. As one participant noted, it is not always convenient to turn on music when you are experiencing pain. But the Veterans who received MI described changes that may have longer lasting effects. As one MI participant said, *“it’s kind of an awakening, you know, it’s like I’m no longer the person I started to be in the study. I ended up transitioning to this, not only this other person but this other identity that, you know, almost like, not an alter ego but a better side of myself”* (MI 284).

### Strengths and limitations

To address some of the methodological limitations, Veterans were interviewed by research team members they had not interacted with during the study. All transcripts were coded by a minimum of two people and discussed in a larger team for accuracy. The interviews were limited to Veterans who agreed to participate and who remained for the full study. Despite efforts to reach participants, we were unable to obtain further feedback from participants who declined the interview or were lost to follow-up. This may lead to more positive statements and endorsement if the Veterans who agreed to interview perceived the intervention as helpful.

## Conclusion

The Veterans who participated in FAMILIA found telehealth-delivered ML or MI sessions to be acceptable and reported benefits and challenges of study participation. The biopsychosocial model suggests that the causes and outcomes of many illnesses result from the interplay of biological, psychological, and social-environmental factors. Effective chronic pain management recognizes and addresses all of these interconnected dimensions. FAMILIA participants reported experiencing biopsychosocial benefits, supporting the use of music therapy as a viable approach to chronic pain management. This aligns with the VA’s recommendation for a comprehensive, interdisciplinary model of care that integrates the biopsychosocial framework across the continuum of chronic pain treatment. FAMILIA findings highlight potential mechanisms to be tested in future studies, including level of absorption in the experience as indicated by frequency of independent listening, presence of a therapist, and use of imagery to enhance absorption.

## Data Availability

Due to privacy and ethical considerations, final data sets resulting from this research will not be shared outside the Department of Veterans Affairs, except as required under the Freedom of Information Act. Requests to access the datasets should be directed to the corresponding author: Kristin.Story@va.gov.

## Author disclaimer

The opinions contained herein represent the private views of the authors and are not to be construed as official or as reflecting the views, opinions, or policies of the Henry M. Jackson Foundation for the Advancement of Military Medicine, Inc., the Department of Defense, the National Endowment for the Arts, or the U. S. Government. Mention of trade names, commercial products, or organizations does not imply endorsement by the U. S. Government. This material was created free of branding or market affiliations. The authors are operating solely as contributors. This work was authored as part of official duties as an employee of the United States Government and is therefore a work of the United States Government. In accordance with 17 USC. 105, no copyright protection is available for such works under US Law.
