# Supplementary material for: Military veterans’ perspectives on using music to manage chronic pain: themes from the feasibility and acceptability of music imagery and listening interventions for analgesia study
Source: Front Neurol. 2025 Aug 6;16:1613220. doi: 10.3389/fneur.2025.1613220 (PMC12366462; doi:10.3389/fneur.2025.1613220)
Supplement: Supplementary file 1 [file Data_Sheet_1.docx]

**FAMILIA Aim 3 Draft Interview Guide**

**General Questions for all Intervention Participants**

1. Why did you decide to participate in the FAMILIA study?
2. What were your expectations going into the study? Were these expectations met? Why or why not?
3. Did you have a preference about which study arm you were assigned to? (music listening or music imagery) If yes, why?

Probe: Did you get the study arm you hoped for? How did that turn out for you?

1. How do you think you benefitted from participating in the study? (Ask for examples)
   1. How does your neck pain compare to before you started the study?
   2. How does your ability to do things compare to before you started the study?
2. What were the downsides of being in the study? How would you “fix” these?
3. What has changed related to your pain or related symptoms since you started the study?
4. What has stayed the same?
5. Have you felt different in any other way since you started the study? (possible probes: more relaxed, less tense, less anxious)
6. How did you feel immediately after the music listening or music imagery time period was over? Did this change over the 3 months of the study? If yes, how?
7. How did you feel as the study went on and you engaged more with music (through music listening or in the music imagery sessions)? (probe for examples where appropriate)
8. Did you have any difficulties filling out the measurements? If yes, can you describe what the difficulties were?
9. What would you change about the study?

**Questions for Music Imagery Arm**

1. Before participation in FAMILIA, had you ever participated in music therapy from a professional therapist?
2. If yes, how did your experience in this study compare to those other experiences?
3. (If not covered above) Was there anything about working with a music therapist for this study that you didn’t like? (e.g., was it uncomfortable or awkward?)
4. Can you describe any challenges/ benefits to participating in the study virtually rather than in-person?
5. Can you tell me about the process of accessing the virtual platform for sessions? (prompt: any issues with connecting)
6. Can you describe any technology issues you encountered during the Music Imagery sessions? (prompt: related to video, audio, music listening)
7. Describe your relationship with your music therapist (probes: Did you feel comfortable with him/her? How supported did you feel by your therapist?)
8. (If not answered in question above): Did you feel like your therapist was responsive to your needs? (e.g., worked with you to choose music and address your needs in the moment)
9. Did you use other creative mediums during the sessions (writing, movement, playing instruments/singing, drawing/art making)? If so, what was your experience of that? (prompts: did you enjoy it, feel it added anything)
10. Did you use the journal and art supplies in between sessions? If so, can you talk about your experience with that? (prompts: was it helpful, a burden)
11. Would you want a program like this to be offered at this VA? Would you participate in it? Why or why not? Would you recommend it to others? Why or why not?

**Questions for Music Listening Arm**

1. Did the frequency or way that you listen to music change during the course of the study?
2. How was the process of checking in with the study staff? (prompts: Did you feel it was helpful or necessary, how was the frequency and length of checking in?)
3. Were you able to listen to music and keep a record of your music-listening? If not, what prevented this? (possible probes: time constraints, forgot to write it down)
4. Did working with the music therapist to create a playlist and listening to music on your own change your relationship to music in any way? How so? (Probes: Do you choose music differently, listen for different reasons, listen more or less frequently, experience the music differently)
5. Were there any negative parts of music listening? If yes, what were they? How might these be overcome? (Possible probes: Was it a burden to keep a journal/log, check in with study staff, meet with the music therapist to create the playlist?)
6. Will you continue to use music listening in your daily life now that the study is finished? Why or why not?
